# EVERYDAY PROSPECTIVE MEMORY LAPSES AND INFLAMMATION IN OLDER ADULTS

**DOI:** 10.1093/geroni/igac059.2073

**Published:** 2022-12-20

**Authors:** Erin Harrington, Jennifer Graham-Engeland, Martin Sliwinski, Mindy Katz, Karina Van Bogart, Jacqueline Mogle, Richard Lipton, Christopher Engeland

**Affiliations:** Pennsylvania State University, University Park, Pennsylvania, United States; Pennsylvania State University, Pennsylvania State University, Pennsylvania, United States; The Pennsylvania State University, University Park, Pennsylvania, United States; Albert Einstein College of Medicine, New York, New York, United States; Pennsylvania State University, Pennsylvania State University, Pennsylvania, United States; The Pennsylvania State University, University Park, Pennsylvania, United States; Albert Einstein College of Medicine, New York, New York, United States; Pennsylvania State University, University Park, Pennsylvania, United States

## Abstract

Prospective memory (PM) refers to our memory for future intentions, such as attending an appointment or taking medication. Research suggests that PM deficits can distinguish healthy older adults from those in early stages of dementia. However, limited work has examined PM and biological markers associated with pathological memory decline. The current study examined older adults’ everyday PM lapses and inflammation. Older dementia-free adults (n =237, Mage=76.86 years), enrolled in the ongoing Einstein Aging Study, completed a two-week ecological momentary assessment (EMA) as part of the first wave of data collection. Participants provided two blood samples (pre/post EMA) and self-reported daily PM lapses during nightly surveys. Inflammatory levels quantified from blood were averaged and included in regression analyses predicting total number of PM lapses (covarying for: age, education, race, health, BMI, depressive symptoms). Reporting more PM lapses was associated with higher circulating levels of interleukin [IL]-8 (p=.007); no significant associations emerged with C-reactive protein or other circulating or stimulated (ex-vivo) cytokines (IL-1β, IL-4, IL-6, IL-8, IL-10, TNF-a). Gender moderated the observed link between IL-8 and PM lapses (p=.015); specifically, higher levels of IL-8 were associated with more PM lapses among men (95%CI=[0.54, 4.72]) but not women (95%CI=[-1.56, 1.25]). Other researchers that found poor cognitive performance in association with elevated IL-8 have suggested that this relation may be indicative of neurodegeneration and future pathology. Future studies should continue to examine daily PM lapses and inflammation across genders to identify mechanisms through which these constructs may relate to neurodegeneration and dementia risk.

